# Thiol–ene click chemistry: a biocompatible way for orthogonal bioconjugation of colloidal nanoparticles†
†Electronic supplementary information (ESI) available. See DOI: 10.1039/c7sc01447c



**DOI:** 10.1039/c7sc01447c

**Published:** 2017-06-22

**Authors:** Yuan Liu, Weijia Hou, Hao Sun, Cheng Cui, Liqin Zhang, Ying Jiang, Yongxiang Wu, Yanyue Wang, Juan Li, Brent S. Sumerlin, Qiaoling Liu, Weihong Tan

**Affiliations:** a Molecular Science and Biomedicine Laboratory , State Key Laboratory of Chemo/Bio-Sensing and Chemometrics , College of Life Sciences , College of Chemistry and Chemical Engineering , Aptamer Engineering Center of Hunan Province , Hunan University , Changsha , Hunan 410082 , China . Email: qlliu@iccas.ac.cn.; b Center for Research at Bio/Nano Interface , Department of Chemistry , Department of Physiology and Functional Genomics , Health Cancer Center , UF Genetics Institute , McKnight Brain Institute , University of Florida , Gainesville , Florida 32611-7200 , USA . Email: tan@chem.ufl.edu; c George & Josephine Butler Polymer Research Laboratory , Center for Macromolecular Science & Engineering , Department of Chemistry , University of Florida , Gainesville , Florida 32611-7200 , USA . Email: sumerlin@chem.ufl.edu

## Abstract

Bioconjugation based on crosslinking primary amines to carboxylic acid groups has found broad applications in protein modification, drug development, and nanomaterial functionalization. However, proteins, which are made up of amino acids, typically give nonselective bioconjugation when using primary amine-based crosslinking. In order to control protein orientation and activity after conjugation, selective bioconjugation is desirable. We herein report an efficient and cysteine-selective thiol–ene click reaction-based bioconjugation strategy using colloidal nanoparticles. The resulting thiol–ene based aptamer and enzyme nanoconjugates demonstrated excellent target binding ability and enzymatic activity, respectively. Thus, thiol–ene click chemistry can provide a stable and robust crosslinker in a biocompatible manner for bioconjugation of any thiol-containing biomolecule with nanomaterials. This will open more opportunities for applications of thiol–ene reactions and functional colloidal nanoparticles in chemical biology.

## Abstract 2

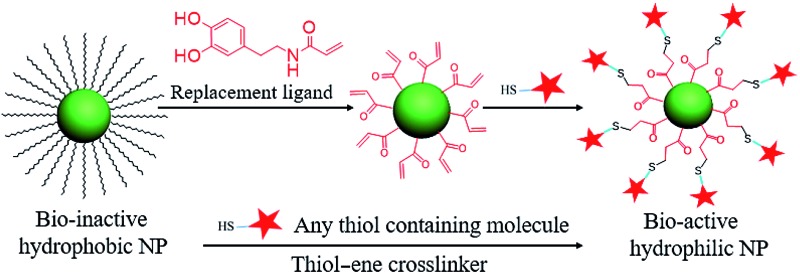
Bioconjugation based on crosslinking primary amines to carboxylic acid groups has found broad applications in protein modification, drug development, and nanomaterial functionalization.

## Introduction

With their unique physicochemical properties, hydrophobic colloidal nanoparticles have broad application in biochemistry,^1^ in areas such as bioimaging, drug delivery, cancer therapy, and enzyme mimicry.^2–5^ On the other hand, the lack of biocompatibility has, to some extent, limited their applications.^6^ To overcome this obstacle, hydrophobic colloidal nanoparticles must typically first be transferred to an aqueous phase, followed by surface functionalization through 1-ethyl-3-(3-dimethylaminopropyl)carbodiimide hydrochloride (EDC)/*N*-hydroxysuccinimide (NHS) coupling, or the Michael addition of a nucleophile to a maleimide.^7,8^ However, EDC/NHS coupling usually has low crosslinking efficiency, and while the maleimide reaction is rapid and has been widely used for antibody drug conjugates, the succinimide linkage of the maleimide addition product is susceptible to hydrolysis.^9,10^ Therefore, even though nanomaterial bioconjugates have enjoyed success, the chemistry of nanoparticle–biomolecule linkage still determines their applications in biochemistry.

“Click” chemistry includes a class of biocompatible reactions that are often employed to join substrates to biomolecules in a quick, selective, and high-yielding manner.^11^ With its efficiency and selectivity, click chemistry is a powerful tool in the field of biomolecular labeling, cell surface modification and drug development.^12,13^ Many chemical ligations have been employed to fulfill the demands of bioorthogonal reactions, including copper-catalyzed azide–alkyne reactions.^14,15^ However, no azides or alkyne functional groups are found among native biomolecules, thus making it necessary to specially introduce these groups into proteins or DNA. Compared to the azide–alkyne reaction, we suggest that the thiol functional group of cysteine-containing proteins makes bioconjugation more readily achievable through a thiol–ene click reaction.^16,17^ So far, the thiol–ene click reaction has been extensively studied in synthetic methodologies, nanoparticle surface modification, and polymerization.^18,19^ But those studies mainly focused on organic systems, which limit their applications in biochemistry.^20,21^ Thus, the thiol–ene reaction-based bioconjugation of colloidal nanoparticles will open up more opportunities for applications of the thiol–ene reaction and functional colloidal nanoparticles.

**Scheme 1:**
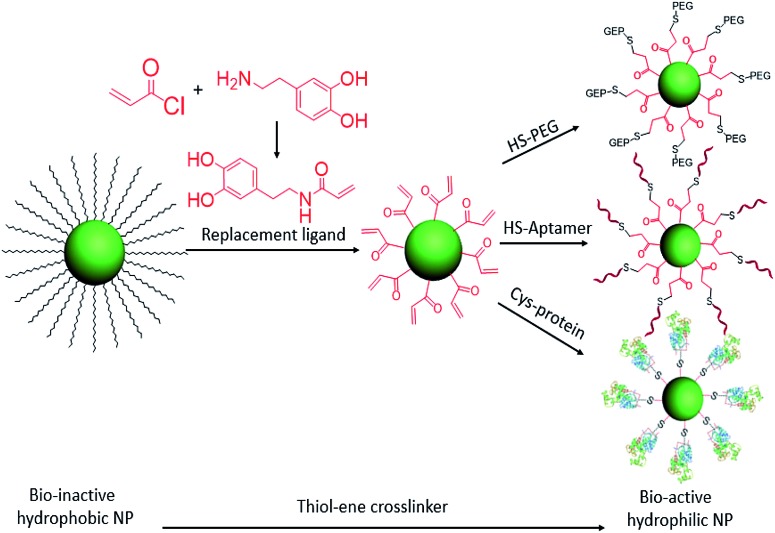
Ligand exchange of hydrophobic colloidal nanoparticles and subsequent thiol–ene click reaction for bioconjugation.

At the interface of biology and nanomaterials, bionanotechnology aims to utilize the unique properties of nanomaterials within a biological context to overcome the problems associated with systemic administration of drugs and contrast agents.^22–24^ We have previously reported a facile ligand exchange method for colloidal nanoparticle surface functionalization.^5,7^ Now, with the advantages of the click reaction, we have designed a cysteine-selective and robust crosslinker and applied it to thiol–ene click reactions for the bioconjugation of nanomaterials. Specifically, the double bond group was modified on the replacement ligand and then anchored on the hydrophobic colloidal nanoparticle surface *via* ligand exchange. With this advantage, any thiol-containing biomolecules can be conjugated on the surfaces of nanoparticles. Here, HS-PEG, HS-DNA, and cysteine-containing horseradish peroxidase are selected to test the thiol–ene crosslinker for bioconjugation of colloidal nanoparticles (Scheme 1).

## Results and discussion

To increase the efficiency of the thiol–ene click reaction, we selected acryloyl chloride, which is a better electrophile than the usual methacryloyl chloride, to incorporate the ene group. Therefore, as shown in Scheme 1, when reacted with dopamine, the product, dopamine acrylamide, as the replacement ligand, can replace the surfactant stabilizer oleic acid to form a robust anchor on the surface of colloidal nanoparticles *via* a five-membered metallocyclic chelate.^25^ Using this method, hydrophobic lanthanide-doped upconversion nanoparticles (UCNPs),^26^ iron oxide nanoparticles,^27^ and manganese oxide nanoparticles^28^ were all synthesized to test the thiol–ene-based crosslinker. As an example, NaYF_4_ (Yb 30%, Er 2%) was selected to study optical properties and biocompatibility after bioconjugation with HS-PEG, HS-DNA, and cysteine-containing horseradish peroxidase.

**Fig. 1:**
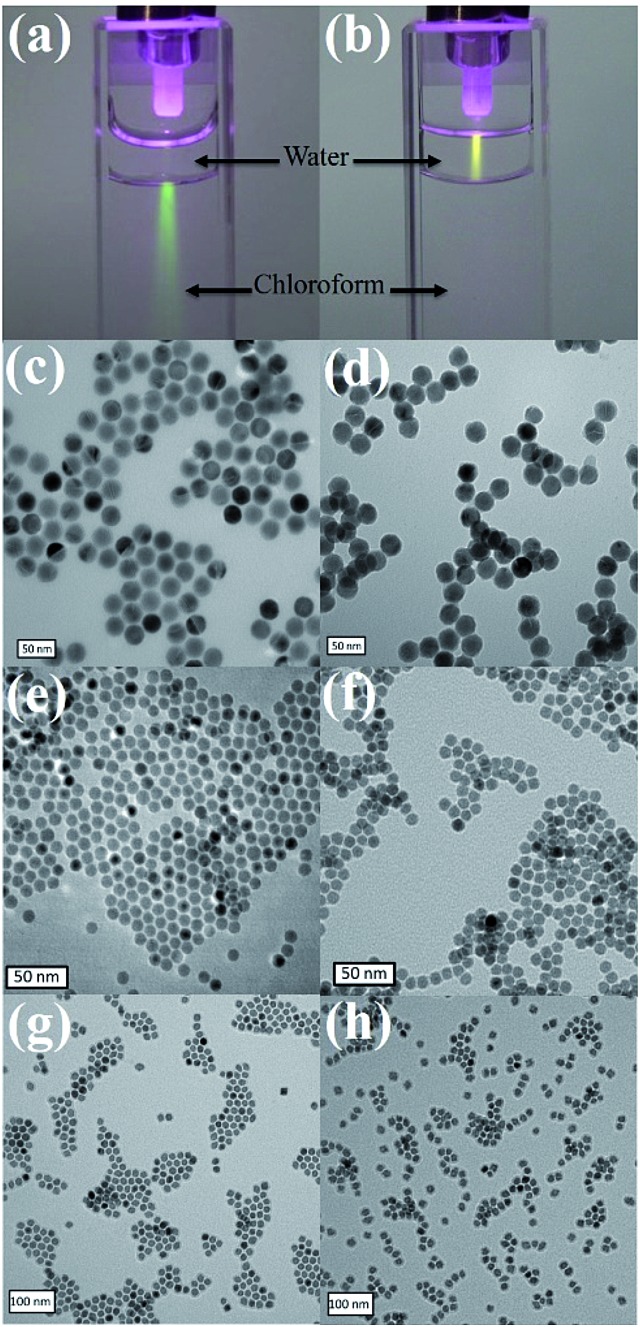
Photographs of the UCNPs in chloroform (a) before ligand exchange and water (b) after thiol–ene crosslinking with HS-DNA under 980 nm laser illumination. TEM images of UCNPs in hexane (c) and water (d), iron oxide in hexane (e) and water (f), and manganese oxide in hexane (g) and water (h), before ligand exchange and after thiol–ene crosslinking with HS-DNA.

^13^C- and ^1^H-NMR spectroscopy demonstrated that dopamine acrylamide was successfully synthesized (Fig. S1 and S2, ESI†). Both the colloidal nanoparticles and the replacement ligand could be dissolved in tetrahydrofuran (THF). Thus, ligand exchange was conducted in THF as a single-phase system at 40 °C for 3 h. Infrared (IR) spectra of UCNPs showed that the characteristic peaks of amide C=O and phenol C–O appeared after ligand exchange, indicating successful immobilization of the required acrylamide moities (Fig. S3, ESI†). Then, either HS-PEG_1000_ or HS-DNA, after reducing the disulfide bond by 1,4-dithiolthreitol (DTT) and purifying through a desalting column, was dissolved in water and mixed with dopamine acrylamide functional UCNPs in THF for 3 h. Triethylamine was used as a catalyst to promote the thiol–ene click reaction. Upon irradiation with a 980 nm laser, a bright luminescence beam was observed when UCNP–S-DNA was dispersed in water (Fig. 1a and b). No obvious change was observed in the photoluminescence spectra of UCNPs after conjugation with HS-PEG_1000_ or HS-DNA upon excitation at *λ* = 980 nm (Fig. S4, ESI†). Transmission electron microscopy (TEM) revealed that the monodispersed UCNPs, iron oxide NPs, and manganese oxide NPs retained their shape and size in the aqueous phase after conjugation with HS-PEG_1000_ and HS-DNA (Fig. 1). The zeta-potential of UCNPs after conjugation with HS-PEG showed almost neutral surface charge (–3.2 mV). Because the DNA oligonucleotide is negatively charged, UCNP–S-DNA showed negative surface charge (–25.9 mV), as shown in Fig. S5 (ESI†). UCNPs gave significant fluorescence signals at 525 nm after linking with FITC-modified HS-PEG_3400_, indicating a successful covalent thiol–ene conjugation reaction (Fig. S6, ESI†).

Thermogravimetric analysis (TGA) was used to examine the thiol–ene click crosslinking efficiency. Assuming the iron oxide nanoparticles (12 nm) are spherically shaped and using the density of iron oxide, the average mass of a single iron oxide nanoparticle was calculated as 4.52 × 10^–18^ g. Based on TGA curves of dopamine acrylamide modified iron oxide nanoparticles before and after thiol–ene crosslinking with HS-PEG (Fig. S7 and S8, ESI†), the number of dopamine acrylamides was calculated as 1556 and the number of PEG chains after thiol–ene crosslinking was calculated as 984.^29,30^ Thus a 63.23% of thiol–ene crosslinking efficiency was achieved. To rule out the possibility of physical absorption of PEG on iron oxide nanoparticles surface, control experiments were conducted and no obvious physical absorption was found (Fig. S9, ESI†).

**Fig. 2:**
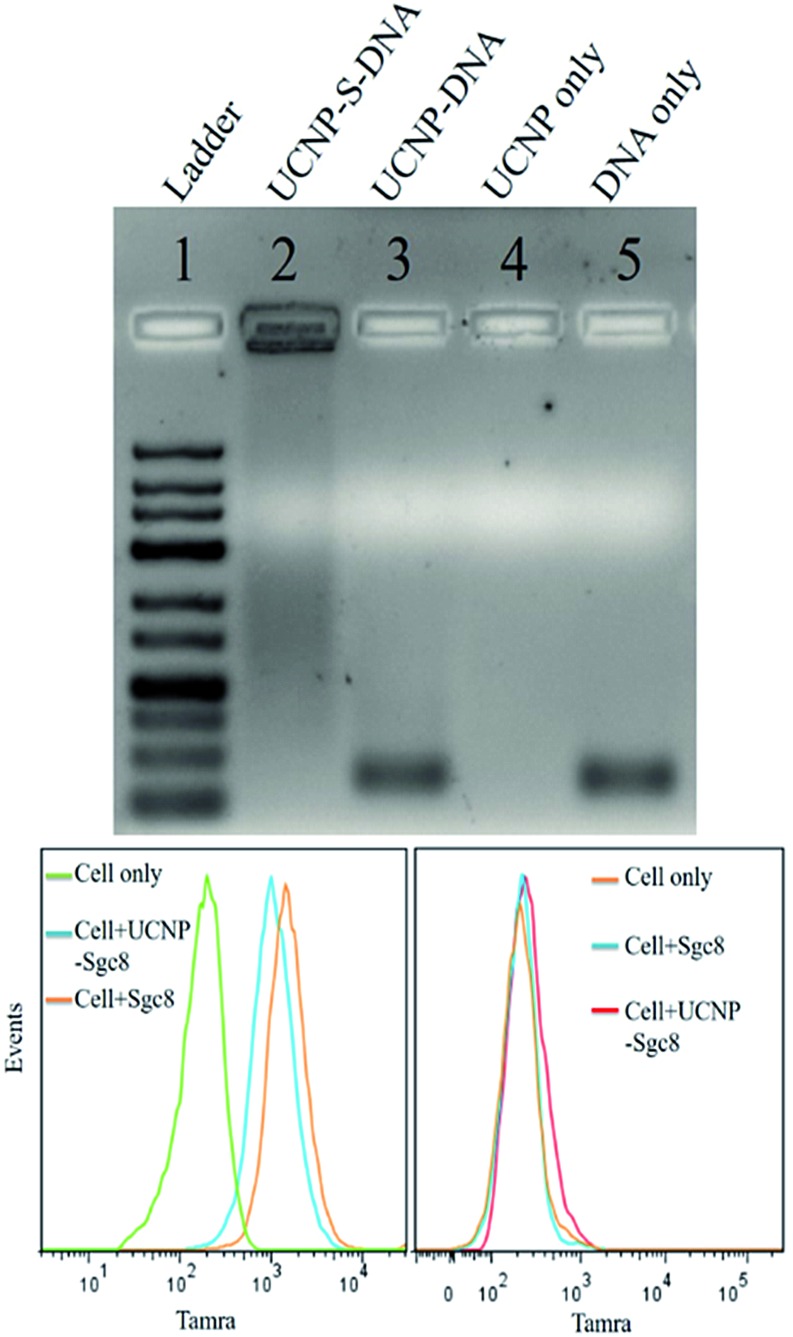
(Top) Stability test of UCNPs thiol–ene click conjugation by agarose gel (lane 1 is ladder. Lane 2 is UCNP–DNA covalent conjugation *via* thiol–ene click chemistry. Lane 3 is UCNP–DNA noncovalent conjugation without thiol–ene click chemistry. Lane 4 is UCNPs only. Lane 5 is DNA only); (bottom) flow cytometry histograms of CEM (left) and Ramos (right) cells incubated with aptamer and UCNP–S-aptamer.

The stability of thiol–ene adducts is a significant factor contributing to their applications in bioconjugate chemistry. Therefore, we used agarose gel electrophoresis to study the stability of the thiol–ene crosslinker with UCNPs. Four parallel samples, including UCNP–S-DNA covalent conjugation *via* the thiol–ene crosslinker, UCNP–DNA noncovalent conjugation without the thiol–ene crosslinker, UCNPs alone, and DNA alone, were prepared for agarose gel electrophoresis. Based on agarose gel imaging (Fig. 2 top), DNA only showed a band at its position. Only UCNPs alone had no band at all. In lane 3, UCNP–DNA refers to UCNPs that were not subjected to the ligand exchange process but contained only physically adsorbed HS-DNA. A very strong band at the free DNA position was observed. This band is attributed to the release of physically adsorbed DNA on the nanoparticle surface under electrophoresis. Without covalent conjugation, physically adsorbed DNA on the nanoparticle surface is not stable under electrophoresis conditions. However, a band was observed in lane 2, which was very strong and did not move. This immobility could be explained by the covalent linkage of DNA on the nanoparticle surface *via* the thiol–ene crosslinker and the large size of UCNPs (28 nm) under electrophoresis. Thus, it was concluded that thiol–ene click chemistry could provide a stable and robust crosslinker.

Having demonstrated a robust thiol–ene crosslinker, we further studied the biomedical applications of UCNPs using HS-aptamer. Thiol-modified Sgc8 aptamer labeled with carboxytetramethylrhodamine (TAMRA) was conjugated on the UCNP surfaces *via* thiol–ene click chemistry to test the aptamer's binding ability to target cancer cells (Table S1, ESI†). Aptamer Sgc8 can bind with the membrane protein PTK7, which is highly expressed on CEM cells.^31,32^ Ramos cells with less expression of PTK7 were used as a negative control. As shown by flow cytometry histograms in Fig. 2 (bottom), an obvious shift was observed for CEM cells, while only a negligible shift was observed for Ramos cells, indicating the excellent target binding ability of this UCNP–S-aptamer.

**Fig. 3:**
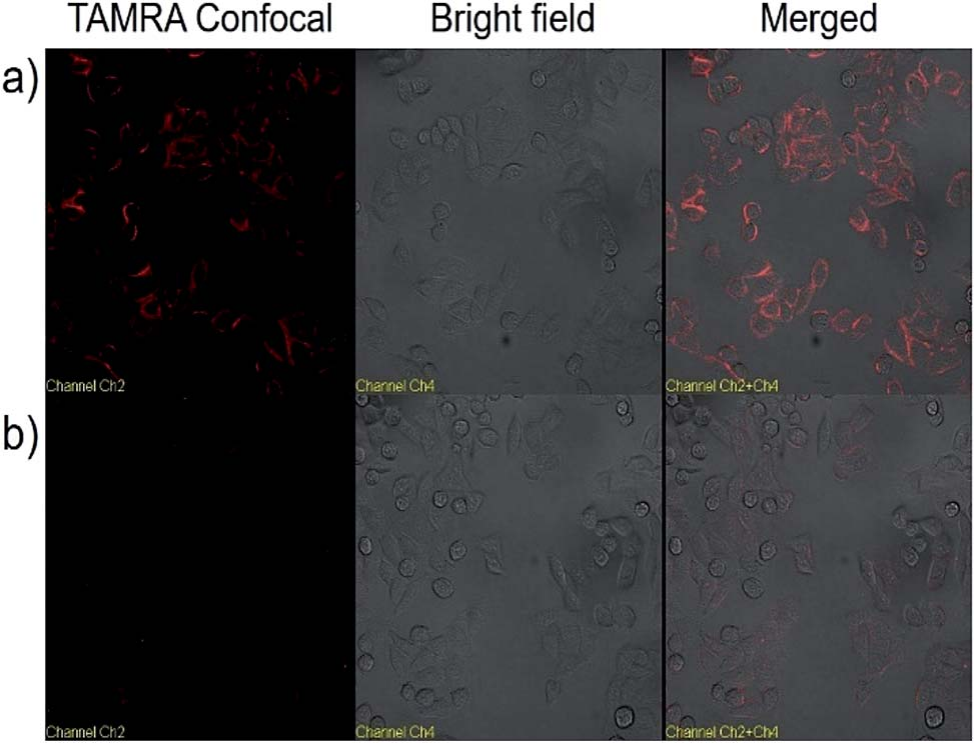
Confocal microscopy images of HeLa cells treated with TAMRA labeled UCNP–Sgc8 (a), and TAMRA labeled UCNP–T20 (b) in Dulbecco's modified Eagle's medium.

We next explored the targeted binding of UCNP–Sgc8 with the human cervical carcinoma (HeLa) cell line, using TAMRA-labeled UCNP–Sgc8 and TAMRA-labeled UCNP–T20. The cellular binding of UCNP–Sgc8 complex was then monitored using confocal laser scanning microscopy (CLSM), as shown in Fig. 3a. Significant red fluorescence was observed after the cells were treated with TAMRA-labeled UCNP–Sgc8 (25 μg mL^–1^). In contrast, only negligible fluorescence was observed when the cells were treated with TAMRA-labeled UCNP–T20. This targeting specificity of Sgc8 aptamer matches well with the results from the flow cytometry as shown in Fig. 2.

**Fig. 4:**
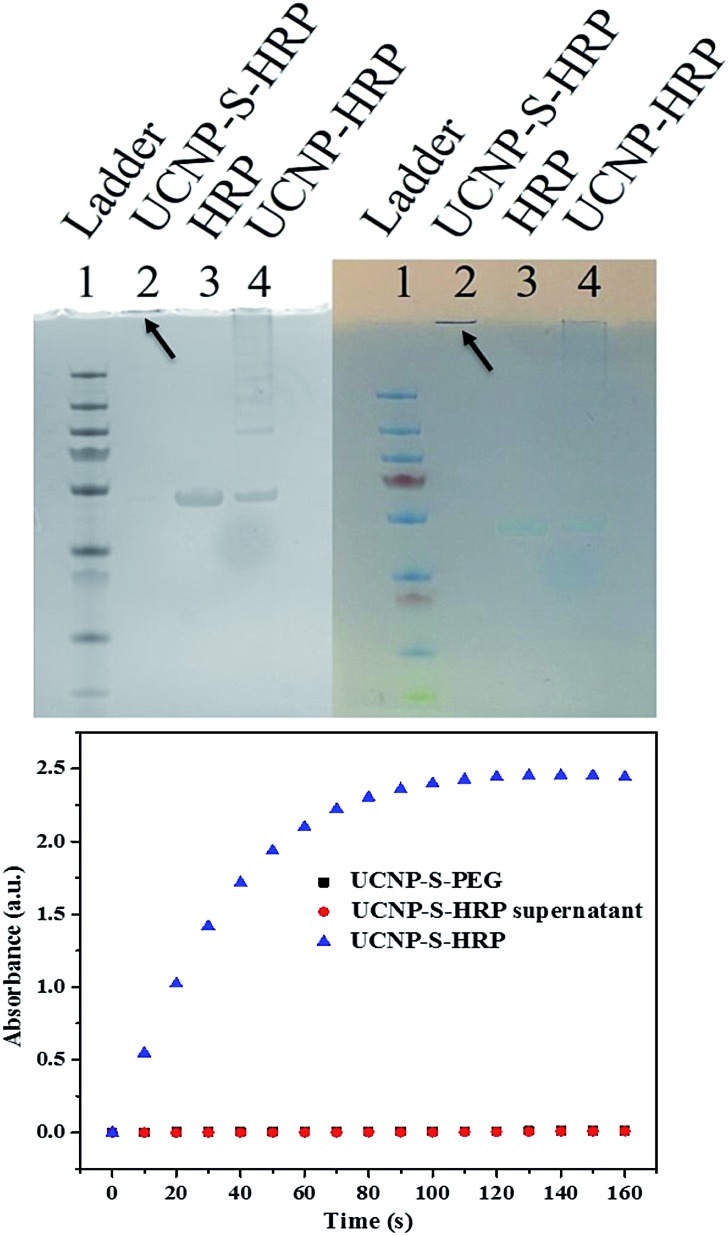
(Top) SDS-PAGE gel of UCNP–S-HRP (2), HRP (3) and UCNP–HRP (4) under UV-light (left) and natural light (right); (bottom) enzymatic activity of UCNP–S-HRP *via* thiol–ene crosslinking, UCNP–S-PEG, and HRP (red dots are superimposed on black squares).

Protein bioconjugation with nanomaterials is a powerful reaction in biochemistry and medicine.^33^ However, the control of protein orientation on the nanoparticles, which is essential in catalysis, delivery, and therapy, necessitates selective bioconjugation.^34–36^ Cysteine is an ideal residue for the chemical modification of proteins based on the unique reactivity of the thiol group and low abundance of cysteine residues in natural proteins. Therefore, cysteine-selective conjugation for bionanoconjugates is desired.^9,10^ In order to study nanoparticle–protein conjugation, horseradish peroxidase (HRP) was selected for conjugation with UCNPs. HRP has 8 cysteines which form 4 disulfide bonds. Before thiol–ene crosslinking, the disulfide bonds of HRP were reduced by treatment with DTT. The reduced HRP was purified by a desalting column before crosslinking with dopamine acrylamide-functionalized UCNPs. Triethylamine (TEA) was added as a catalyst to promote the thiol–ene click reaction. The resultant UCNP–S-HRP conjugates were then analyzed by SDS-PAGE (sodium dodecyl sulfate polyacrylamide gel electrophoresis). As shown in Fig. 4 (top), to obtain a better comparison, the gel pictures were taken under UV-light (left) and natural light (right). HRP without reduction or TEA catalysis was physically adsorbed on the surfaces of UCNPs and showed an HRP band and a long tail band (lane 4) under electrophoresis. In contrast, the UCNP–S-HRP conjugated *via* thiol–ene crosslinking gave a single band (lane 2), but did not move owing to the large size of UCNPs, indicating that a stable and robust thiol–ene linkage had occurred between UCNPs and HRP.

As a result of structural disturbance, enzymatic activity may be affected by conjugation with nanomaterials, because it is dependent on active sites with access to the environment.^31^ HRP is an enzyme which can catalyze the oxidation of 3,3′5,5′-tetramethylbenzidine (TMB) in the presence of hydrogen peroxide. Thus, we further studied the catalytic activity of HRP after conjugation with UCNPs to form UCNP–S-HRP *via* thiol–ene click reaction. As shown in Fig. 4 (bottom), UCNPs alone (black square) show no catalytic activity. UCNP–S-HRP was washed 4 times after conjugation until all free HRP enzyme was removed and no catalytic activity was observed in the supernatant. However, under the same conditions, we observed excellent catalytic activity from UCNP–S-HRP in the presence of TMB (blue triangles). For HRP enzyme activation, its substrate TMB must react with the active sites and finally release the product. Therefore, the cysteine-selective conjugation with dopamine acrylamide-functionalized UCNPs *via* the thiol–ene click reaction had no deleterious effect on the active site of HRP enzyme after conjugation.

## Conclusion

In conclusion, we have developed a thiol–ene based bioconjugation strategy in a biocompatible manner for colloidal nanoparticle-based bioconjugates, including iron oxide, manganese oxide, and UCNP, and tested the crosslinker by HS-PEG pegylation, HS-aptamer labeling, and enzyme immobilization. Gel electrophoresis demonstrated that the thiol–ene crosslinker is stable and robust. Moreover, the stable and robust thiol–ene linkage between dopamine acrylamide-functionalized UCNPs and aptamer or HRP did not affect the binding ability of aptamer to its target cells or the catalytic activity of HRP enzyme. Bioconjugates based on reactions with nanomaterials have enormous potential in such fields as biology and materials science. In particular, the superior selectivity and stability of the thiol–ene adduct will enable engineering of multifunctional nanomaterial bioconjugates, making this a powerful tool with broad applications in biosensing, bioanalysis, bioimaging, drug delivery, and theranostics.
