# Supplementary material for: Thiol–ene click chemistry: a biocompatible way for orthogonal bioconjugation of colloidal nanoparticles
Source: Chem Sci. 2017 Jun 22;8(9):6182–7. doi: 10.1039/c7sc01447c (PMC5628335; doi:10.1039/c7sc01447c)
Supplement: Supplementary file 1 [file SC-008-C7SC01447C-s001.pdf]

Electronic Supplementary Material (ESI) for Chemical Science.
This journal is © The Royal Society of Chemistry 2017

Supporting Information:

Thiol-ene Click Chemistry: A Biocompatible Way for Orthogonal Bioconjugation of Colloidal Nanoparticles

Yuan Liu, † Weijia Hou,† Hao Sun,§ Cheng Cui,† Liqin Zhang,† Ying Jiang,† Yongxiang Wu,‡ Yanyue Wang†, Juan Li,‡ Brent S. Sumerlin,§ Qiaoling Liu,‡ Weihong Tan*,†, ‡

†Center for Research at Bio/Nano Interface, Department of Chemistry and Department of Physiology and Functional Genomics, Shands Cancer Center, UF Genetics Institute and McKnight Brain Institute, University of Florida, Gainesville, Florida 32611-7200, United States

‡Molecular Science and Biomedicine Laboratory, State Key Laboratory of Chemo/Bio-Sensing and Chemometrics, College of Biology and College of Chemistry and Chemical Engineering, Hunan University, Changsha, 410082, China

§George & Josephine Butler Polymer Research Laboratory, Center for Macromolecular Science & Engineering, Department of Chemistry, University of Florida, Gainesville, Florida 32611-7200, USA

Contents

Materials.....3

Synthesis of *N*-(2-[3,4-dihydroxyphenyl] ethyl) acrylamide (dopamine acrylamide).....3

Synthesis of upconversion nanoparticles ($NaYF_4$).....6

Synthesis of iron oxide nanoparticles.....6

Synthesis of iron oleate.....6

Synthesis of iron oxide nanoparticles.....7

Synthesis of MnO Nanoparticles.....7

Synthesis of manganese oleate.....7

Synthesis of manganese oxide.....7

Acrylation of hydrophobic nanoparticles.....8

Pegylation of acrylated nanoparticles.....8

Characterization techniques.....8

Synthesis of thiol-modified Sgc8 aptamer.....9

UCNP conjugation with HS-DNA.....9

UCNP-DNA without thiol-ene crosslinking.....9

UCNP conjugation with Horseradish Peroxidase (HRP) enzyme.....10

UCNP-HRP without thiol-ene crosslinking.....10

Agarose gel electrophoresis.....10

SDS-page gel electrophoresis.....10

Target binding test with flow cytometry.....11

Internalization study.....11

Enzymatic activity test of UCNP-S-HRP.....11

Paper table and figures.....12

References.....19

Materials

All reagents used were purchased from Sigma-Aldrich, unless otherwise stated. D_6-DMSO was purchased from Cambridge Isotopes. Sodium oleate was purchased from Tokyo Chemical Industry. HS-PEG-FITC was purchased from Nanocs Inc. DNA synthesis reagents were acquired from Glen Research Corp. and ChemGenes Corp. All oligonucleotides were synthesized and purified in our group. The water used was purified on a Milli-Q Biocell System. SDS-PAGE analysis was performed on precast NuPAGE® Novex 4-12% bis-tris gels according to the manufacturer's protocol and stained with SYBR® Gold and GelCode™ Blue Safe Protein Stain.

Synthesis of *N*-(2-[3,4-dihydroxyphenyl] ethyl) acrylamide (dopamine acrylamide)

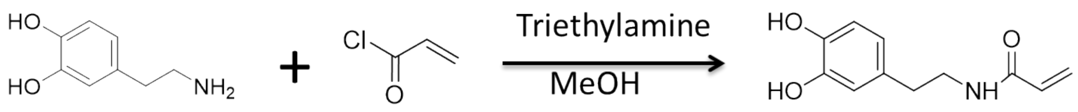

Dopamine hydrochloride (2 g, 10.6 mmol) and triethylamine (1.46 mL, 10.6 mmol) were dissolved in 20 mL anhydrous methanol in a 100 mL flask. The flask was cooled in an ice bath for 30 min. Acryloyl chloride (1.22 mL, 1.26 mmol) in 1 mL of tetrahydrofuran, and triethylamine (2.20 mL, 15.9 mmol) in 2 mL of methanol were added dropwise to the flask. Then, the ice bath was removed and the reaction mixture was stirred for 2 hours at room temperature. Solvent was removed using rotary evaporation. Ethyl acetate (50 mL) was added to dissolve the residue. Hydrochloric acid (15 mL, 1M) and brine (15 mL) were used to wash the product. The organic layer was collected, dried with anhydrous magnesium sulfate, and then filtered and concentrated by evaporation. The product was recrystallized with ethyl acetate to give a white solid in 50% yield.

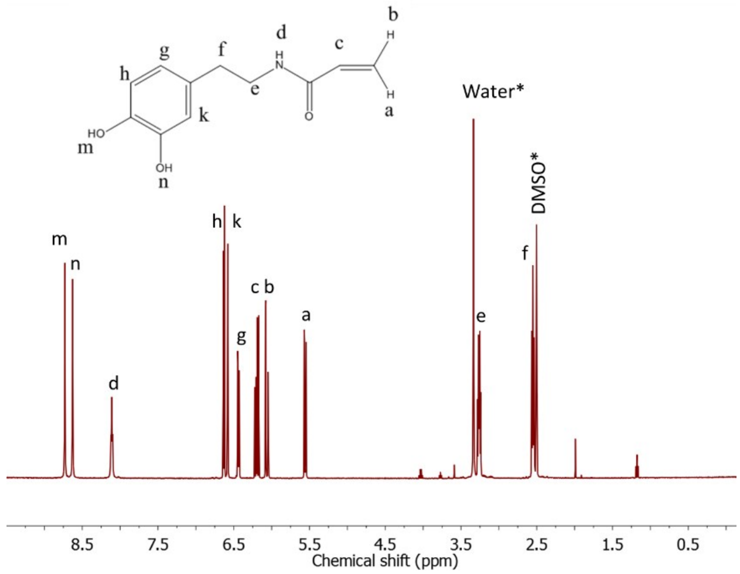

Figure S1. ^{1}H-NMR (500 MHz, d_6-DMSO, δppm) of dopamine acrylate.

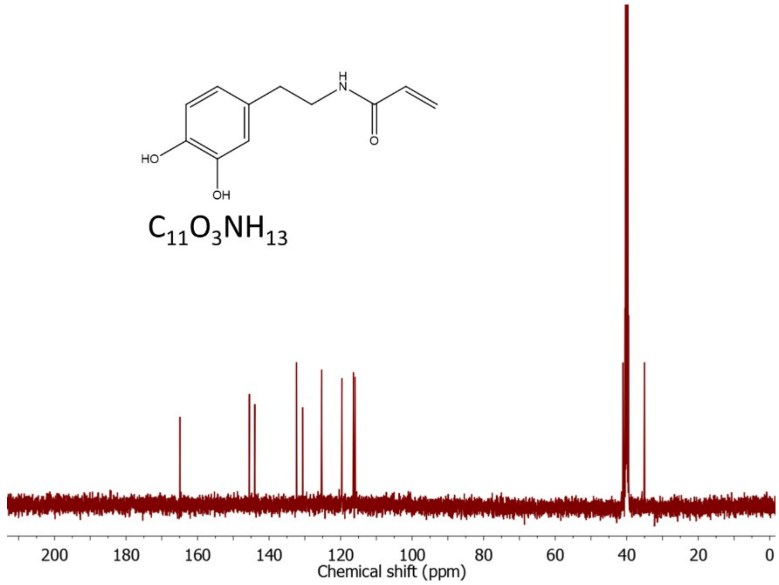

Figure S2. ^{13}C-NMR (125MHz, d_6-DMSO, δppm) of dopamine acrylate.

Synthesis of upconversion nanoparticles ($NaYF_4$)

$NaYF_4$ (Yb 30%, Er 2%) nanoparticles were synthesized using a procedure similar to the previously published method.[1] Typically, $Y(CH_3CO_2)_3$ (0.68 mmol), $Yb(CH_3CO_2)_3$ (0.3 mmol), and $Er(CH_3CO_2)_3$ (0.02 mmol) were added to a 100 mL round-bottomed flask containing oleic acid (7.5 mL) and 1-octadecene (17.5 mL). The resulting mixture was heated to 150 °C and kept for 0.5 h. After cooling to room temperature, a methanol solution (6 mL) containing NH_4F (4 mmol) and NaOH (2.5 mmol) was added. The mixture was stirred for 0.5 h at 50 °C, followed by heating to 100 °C to remove the methanol. Finally, the crude product was heated to 290 °C and maintained for 2 h under argon flow. The resulting nanoparticles were washed with hexane and ethanol and redispersed in tetrahydrofuran (THF).

Synthesis of iron oxide nanoparticles.

Synthesis of iron oleate.

Iron oleate was synthesized using a modified literature method.[2] Typically, 10.8 g iron (III) chloride ($FeCl_3{\cdot}6H_20$, 40 mmol) and 36.5 g sodium oleate (120 mmol) were dissolved in a mixed solvent composed of 80 mL ethanol, 60 mL distilled water and 140 mL hexane. The resulting solution was heated to 60 °C and refluxed for 4 hours. When the reaction was finished and cooled to room temperature, the upper organic layer containing the iron oleate complex was washed three times with distilled water using a separatory funnel. After removal of hexane, the resulting iron oleate complex was in a waxy solid form.

Synthesis of iron oxide nanoparticles.

Hydrophobic magnetic nanoparticles were synthesized using a modified protocol.[2] Iron oleate 0.9 g (1 mmol) and oleic acid 0.156 g (0.55 mmol) were added to a three-necked flask (25 mL) with a solvent of 1-octadecene (5 g). The reaction mixture was heated to 320 °C at a heating rate of ~18 °C/min. After 1 hour, the reaction mixture was quickly cooled to room temperature by blowing air across the reaction flask. The resulting iron oxide magnetic nanoparticles were

purified with acetone/hexane (precipitation/redispersion) for three rounds. After purification, the product was dispersed in THF for further use.

Synthesis of MnO Nanoparticles.

Synthesis of manganese oleate

Manganese (II) oleate was prepared based on a reported method.[3] In a typical synthesis, 40 mmol of manganese (II) chloride tetrahydrate and 80 mmol oleic acid were mixed together in 200 mL of methanol. Then, 80 mmol of sodium hydroxide dissolved in 200 mL of methanol were added to the above solution dropwise. A red oily precipitate was obtained from a clear colorless mixture. After two hours stirring, the precipitate was washed with water, ethanol, and acetone, then dissolved in hexane. The oily residue was dried with anhydrous $MgSO_4$. Finally, a deep red waxy solid was obtained after evaporating the solvent and drying in vacuum.

Synthesis of manganese oxide

In a typical synthesis, 1 mmol of manganese oleate was dissolved in 5 g of 1-octadecene and degassed at 80 °C for 1 hour to remove the moisture and oxygen. Then the reaction mixture was heated to 270 °C and maintained for 1 hour. The as-prepared nanoparticles were washed with hexane/ethanol and finally dispersed in THF for future ligand exchange.

Acrylation of hydrophobic nanoparticles

Dopamine acrylate (50 mg) was dissolved in 5 mL of THF in a 25 mL three-necked flask and heated to 40 °C. Then 10 mg of hydrophobic nanoparticles (UCNP, iron oxide, or manganese oxide) in 2 mL of THF were added to the flask, and the mixture was incubated for 3 hours. After incubation, the acrylated nanoparticles were washed with ethanol and THF, and finally redispersed in THF.

Pegylation of acrylated nanoparticles

Acrylated nanoparticles (UCNP, iron oxide, manganese oxide) in THF (1 mL, 0.5 mg/mL) were mixed with HS-PEG_{1000} (HS-PEG_{3400}-FITC) in water (200 µL, 20 mg/mL). Then, 20 µL of

triethylamine in THF (5 mg/mL) was added as a catalyst to promote the thiol-ene click reaction. The reaction mixture was incubated for 2.5 hours at room temperature. After incubation, nanoparticles were washed 3 times with water and finally redispersed in water for characterization.

Characterization techniques

Transmission Electron Microscopy (TEM). Imaging was carried out using a Hitachi H-7000 transmission electron microscope at 100 kV. Five microliter samples of colloidal nanocrystals in water were dropped onto a carbon-coated copper grid (Ted Pella) and then dried for TEM.

ζ-Potential. ζ-Potentials were determined at room temperature using a Zetasizer Nano-ZS (Malvern).

^{1}H NMR and ^{13}C NMR were recorded on a Varian Inova2 500 MHz NMR spectrometer.

FT-IR Spectra. Spectra were recorded with a near- and mid-IR spectrometer (a Nicolet Nexus 670) in KBr pellets.

Optical Absorption Spectroscopy. UV−vis absorption spectra were recorded using a Shimadzu UV-1800. Nanocrystals were dissolved in hexane or water for measurement.

Thermogravimetric Analysis. Thermogravimetric analyses were performed under nitrogen with a TGA Q5000. Approximately 5 mg of iron oxide nanoparticles were prepared. The samples were heated from 20 °C to 600 °C at a ramp rate of 10 °C min^{-1}.

Synthesis of thiol-modified Sgc8 aptamer.

The TAMRA-labeled Sgc8 aptamer with disulfide group (detailed sequence, Table 1) was synthesized using an ABI3400 DNA/RNA synthesizer (Applied Biosystems, Foster City, CA, USA). The disulfide-modified, TAMRA-labeled aptamer was deprotected in 3 mL of deprotection solution (ammonium hydroxide, methylamine 50:50) for 30 min at 65 °C. Then, 250 μL 3 M NaCl and 6 mL cold ethanol were used to precipitate the deprotected sequences. The precipitated aptamers were collected by centrifugation and dissolved in 400 μL of triethylammonium acetate

(TEAA) for further purification by reversed-phase HPLC (ProStar, Varian, Walnut Creek, CA, USA) using a C18 column and acetonitrile-TEAA solvent. The purified aptamer was quantified by UV-vis.

UCNP conjugation with HS-DNA

Thiol-modified DNA was first reduced with dithiothreitol (DTT) and then purified by desalting column to obtain HS-DNA. Acrylated nanoparticles (UCNP, iron oxide, or manganese oxide) in THF (800 µL, 0.5 mg/mL) were mixed with HS-DNA (200 µL, 50 µM). Then 20 µL of triethylamine (5 mg/mL) was added to promote the thiol-ene click reaction. The reaction mixture was incubated for 2.5 hours at room temperature. After incubation, UCNP-S-DNA was washed three times with water and finally redispersed in water.

UCNP-DNA without thiol-ene crosslinking

UCNP without dopamine acrylamide was dispersed in THF (800 µL, 0.5 mg/ml) and was mixed with HS-DNA (200 uL, 50 µM) which was not reduced by DTT. The reaction mixture was incubated for 2.5 hours at room temperature. After incubation, UCNP-DNA was washed three times with water and finally redispersed in water.

UCNP conjugation with Horseradish Peroxidase (HRP) enzyme

HRP contains 8 cysteines, a key residue, and 4 disulfide bonds. To reduce the disulfide bond, HRP (250 µL, 1 mg/mL) was mixed with DTT (100 mM) for 15 mins. Then the reduced HRP was purified by desalting column. To conjugate HRP with UCNP, acrylated UCNP in THF (800 µL, 0.5 mg/mL) was mixed with reduced HRP (100 µL, 0.5 mg/mL). Then 20 µL of triethylamine (5 mg/mL) was added to promote the thiol-ene click reaction. The reaction mixture was incubated for 2.5 hours at room temperature. After incubation, UCNP-S-HRP was washed four times with water until no catalytic activity was observed in the supernatant. Finally, UCNP-S-HRP was redispersed in water.

UCNP-HRP without thiol-ene crosslinking

UCNP-HRP without thiol-ene crosslinking sample was prepared using the same conditions as the preparation of UCNP-S-HRP, but without DTT reduction or addition of trimethylamine. The resulting UCNP-HRP was washed 3 times with water and redispersed in water for future use.

Agarose gel electrophoresis

To analyze the thiol-ene crosslinking stability of UCNP and HS-DNA (UCNP-S-DNA), agarose gel (3%) was prepared using agarose (0.9 g), TBE (30 mL, 1X), and EB (5 μL). Agarose gel electrophoresis was conducted at 100 V for 30 min. HS-DNA, UCNP-DNA, and UCNP without thiol-ene crosslinking were used as controls. The gel was washed with deionized water before taking pictures.

SDS-page gel electrophoresis

To analyze the thiol-ene crosslinking stability of UCNP and HRP (UCNP-S-HRP), SDS-PAGE gel (NuPage 4-12% Bis-Tris Gel) electrophoresis was conducted at 200 V for 45 min. UCNP only, HRP only and UCNP-HRP without thiol-ene crosslinking were used as controls. After SDS-PAGE gel electrophoresis, the gel was washed twice with deionized water and then stained with GelCode™ Blue Safe Protein Stain for 30 min. After staining, the gel was washed three times with deionized water before taking pictures.

Target binding test with flow cytometry

To demonstrate the specific target binding ability of UCNP-S-Aptamer to different cell lines, fluorescence measurements were obtained with a FACSAria™ IIu cytometer (Becton Dickinson, San Jose, CA, USA) using a 555 nm laser as excitation source. Samples containing CEM/Ramos cells with a concentration of 10^6 cells/mL were incubated with the desired concentration of UCNP-Sgc8 and Sgc8 aptamer in 200 μL of binding buffer at 4 °C for 30 min. The resulting cells were washed 3 times with washing buffer and redispersed in binding buffer for flow cytometry analysis by counting 10,000 events.

Internalization study

HeLa cells (80k) were planted in a confocal dish in 500uL Dulbecco's modified Eagle's medium (DMEM) supplemented with 100 U/mL aqueous penicillin G, 100 μg/mL streptomycin, 0.4 mM L-Glutamine and 10% FBS, at 37 ºC in 5% CO_2 for 24h to allow 70% confluence. Cells were washed with PBS (pH 7.4) twice before incubating with UCNP-sgc8 (25 μg/mL) in complete DMEM medium for 4h at 37 °C in 5% CO_2. Nuclei were then stained with Hoechst. The medium was then removed and washed twice with DMEM medium and once with PBS. Thereafter, confocal fluorescence images were taken by a Leica TCS SP5 confocal microscope.

Enzymatic activity test of UCNP-S-HRP

To study the enzymatic activity of UCNP-S-HRP, 3,3'5,5'-tetramethylbenzidine (TMB) (Liquid Substrate System for ELISA) was used as substrate for UCNP-S-HRP. UCNP-S-PEG was used as a control. Kinetics activity measurements were obtained on a Shimadzu UV-1800.

Paper table and figures

Table S1: Detailed sequence information of HS-DNA and HS-Aptamer

Name	Sequence
HS-T20-TAMRA:	5'-HS- TTT TTT TTT TTT TTT TTT TT-TAMRA-3'
HS-DNA:	5'-HS- TTT TTT TTT TAT CTA ACT GCT GCG CCG CCG GGA AAA TAC TGT ACG GTT AGA-3'
HS-Aptamer-TAMRA:	5'-HS- TTT TTT TTT TAT CTA ACT GCT GCG CCG CCG GGA AAA TAC TGT ACG GTTAGA-TAMRA-3'

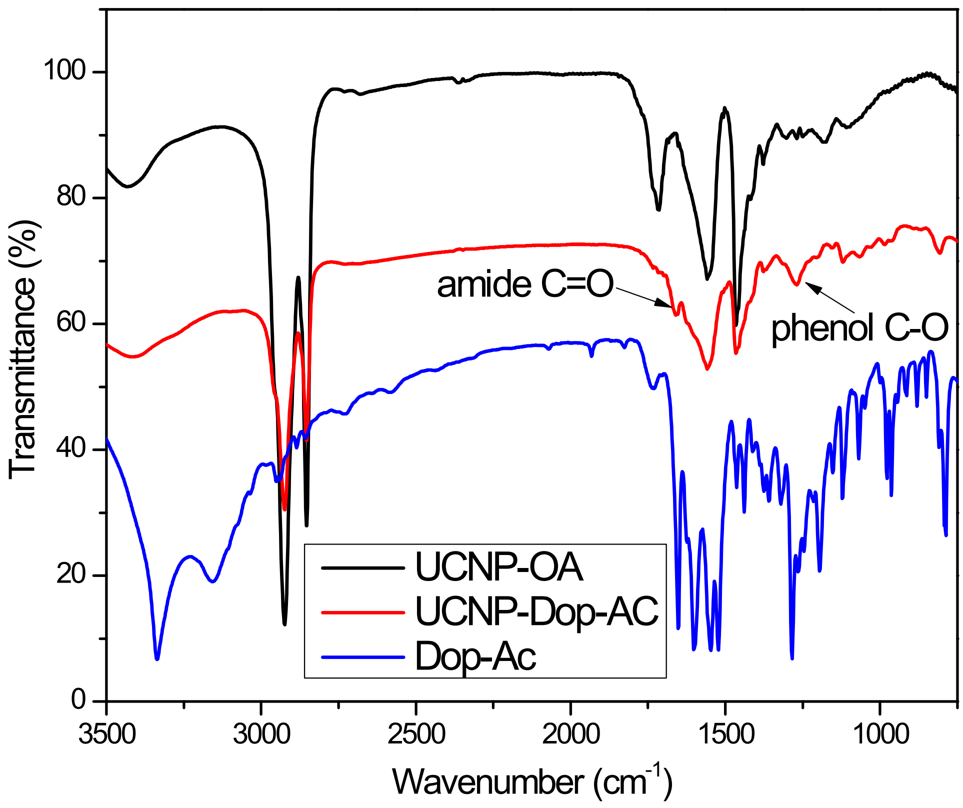

Figure S3. IR spectra of UCNP capped with oleic acid (UCNP-OA, black), dopamine acrylate (Dop-Ac, blue), and acrylated UCNP after ligand exchange (UCNP-Dop-Ac, red).

To prepare the IR samples, UCNP-OA was washed with hexane and ethanol and redispersed in hexane for drying in a vacuum oven. Solid UCNP-OA was collected to prepare a KBr plate for IR measurement. After ligand exchange with Dop-Ac, UCNP-Dop-Ac was washed with tetrahydrofuran and ethanol and redispersed in tetrahydrofuran for drying in a vacuum oven. Solid UCNP-Dop-Ac was collected to prepare a KBr plate for IR measurement. Solid Dop-Ac was used directly to prepare a KBr plate for IR measurement. The IR spectrum of UCNP-Dop-AC indicates peaks of amide C=O (1660 cm^{-1}) and phenol C-O (1270 cm^{-1})[4] as listed above.

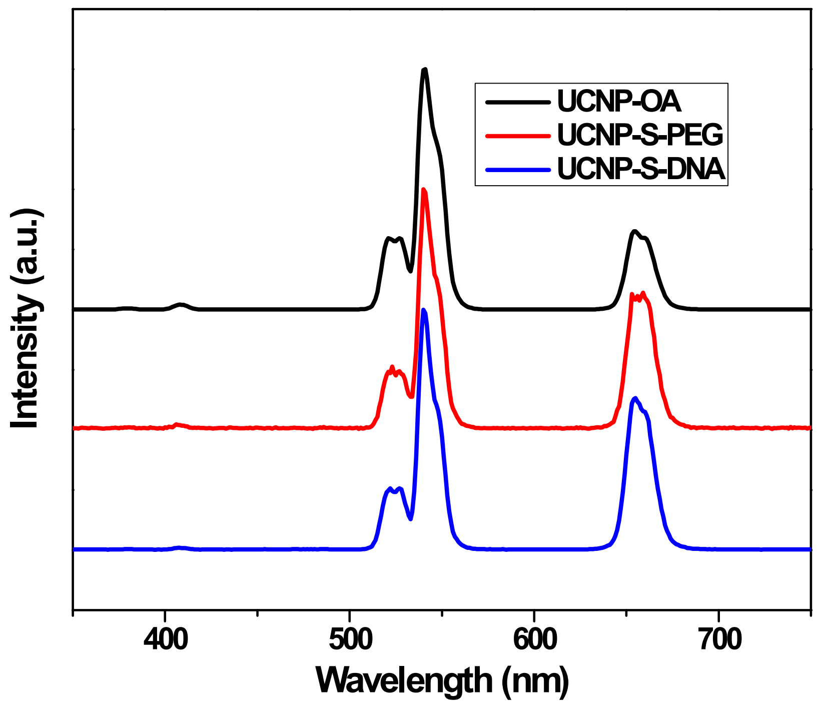

Figure S4. Photoluminescence spectra of UCNP before ligand exchange and after thiol-ene crosslinking with HS-PEG and HS-DNA.

To prepare the samples for photoluminescence measurement, UCNP without ligand exchange was dispersed in hexane. UCNP-S-PEG and UCNP-S-DNA were washed three times with water after thiol-ene crosslinking and dispersed in water, respectively.

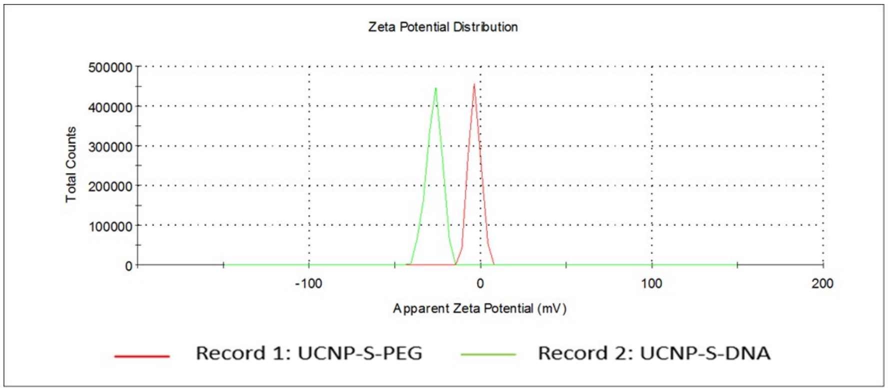

Figure S5. Zeta-potential of UCNP after thiol-ene crosslinking with HS-PEG and HS-DNA. To prepare the samples for zeta-potential measurement, UCNP-S-PEG and UCNP-S-DNA were washed three times with water after thion-ene crosslinking and redispersed in 1 ml of water.

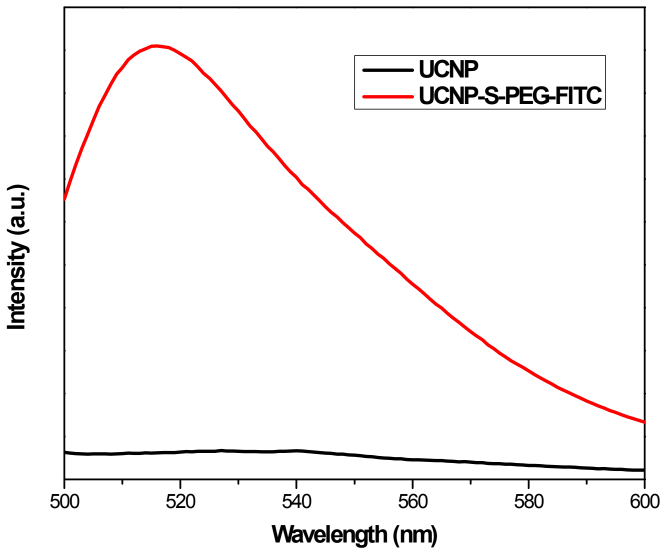

Figure S6. Fluorescence spectrum of UCNP (black) and UCNP-S-PEG-FITC (red) excited at 488 nm, indicating successful pegylation of UCNP.

To prepare the samples for the fluorescence measurement, UCNP was dispersed in hexane. UCNP-S-PEG-FITC was washed three times with water after thiol-ene crosslinking and redispersed in water.

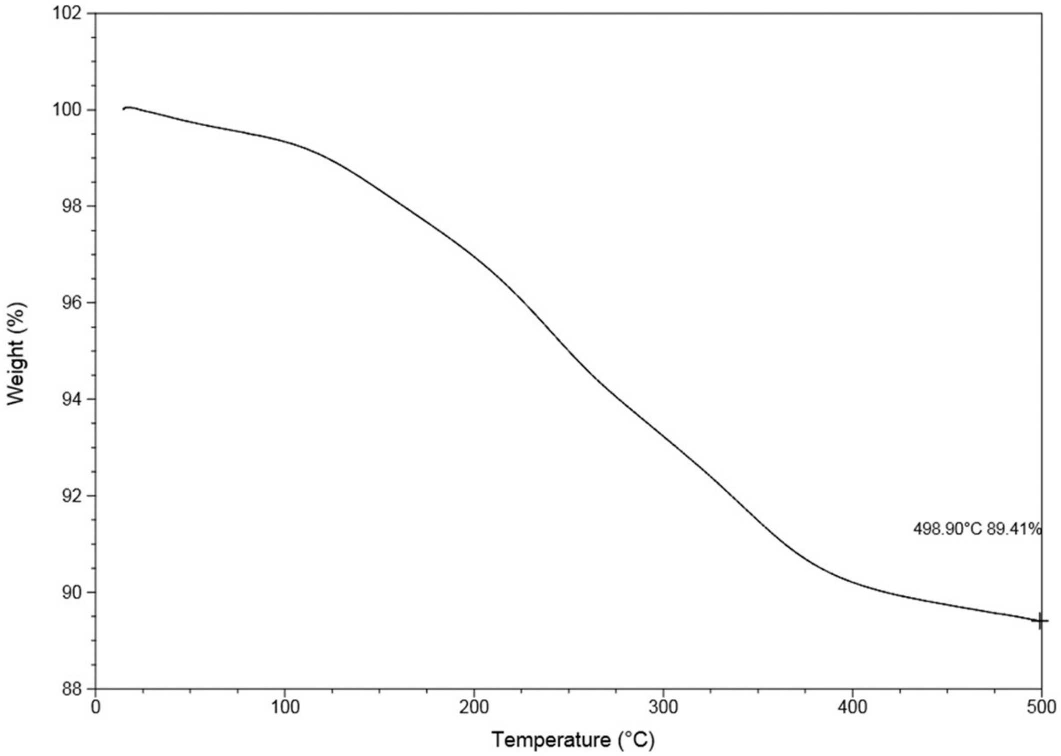

Figure S7. Plot of weight loss of dopamine acrylamide functionalized iron oxide nanoparticles as a function of temperature as measured by themogravimetric analysis.

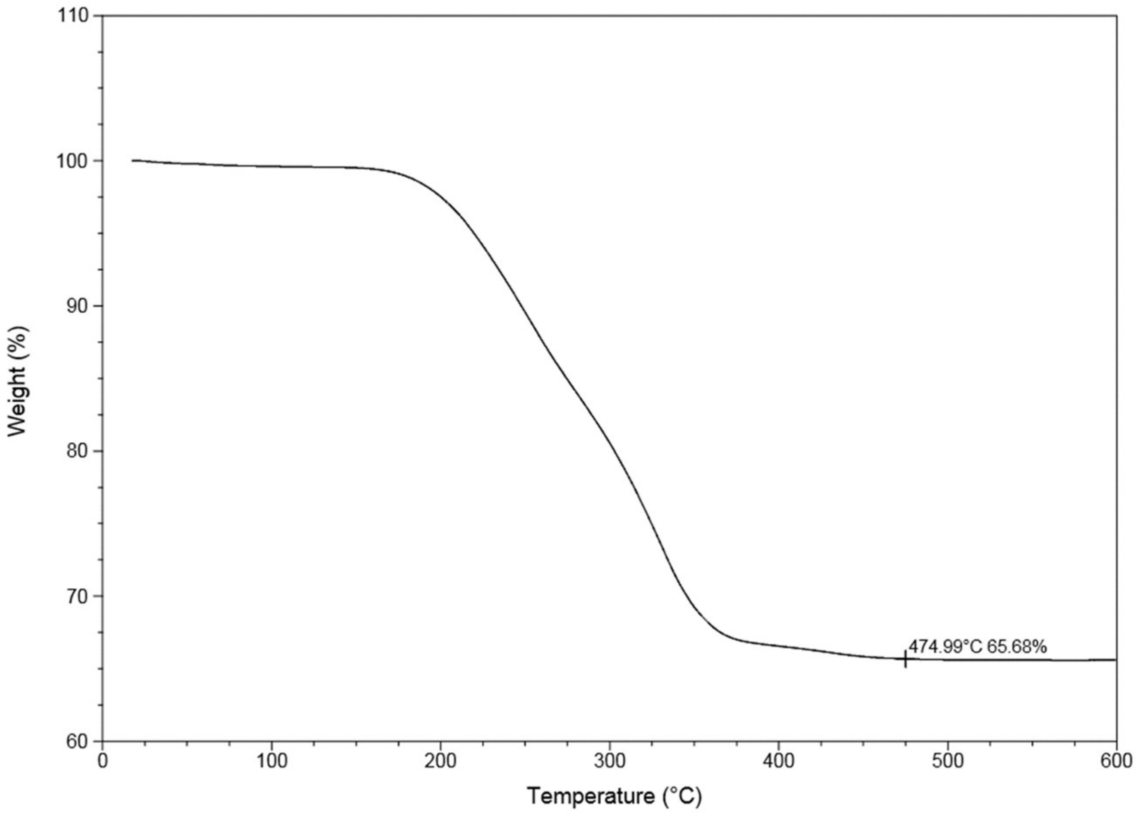

Figure S8. Plot of weight loss of HS-PEG1000 functionalized iron oxide nanoparticles as a function of temperature as measured by thermogravimetric analysis.

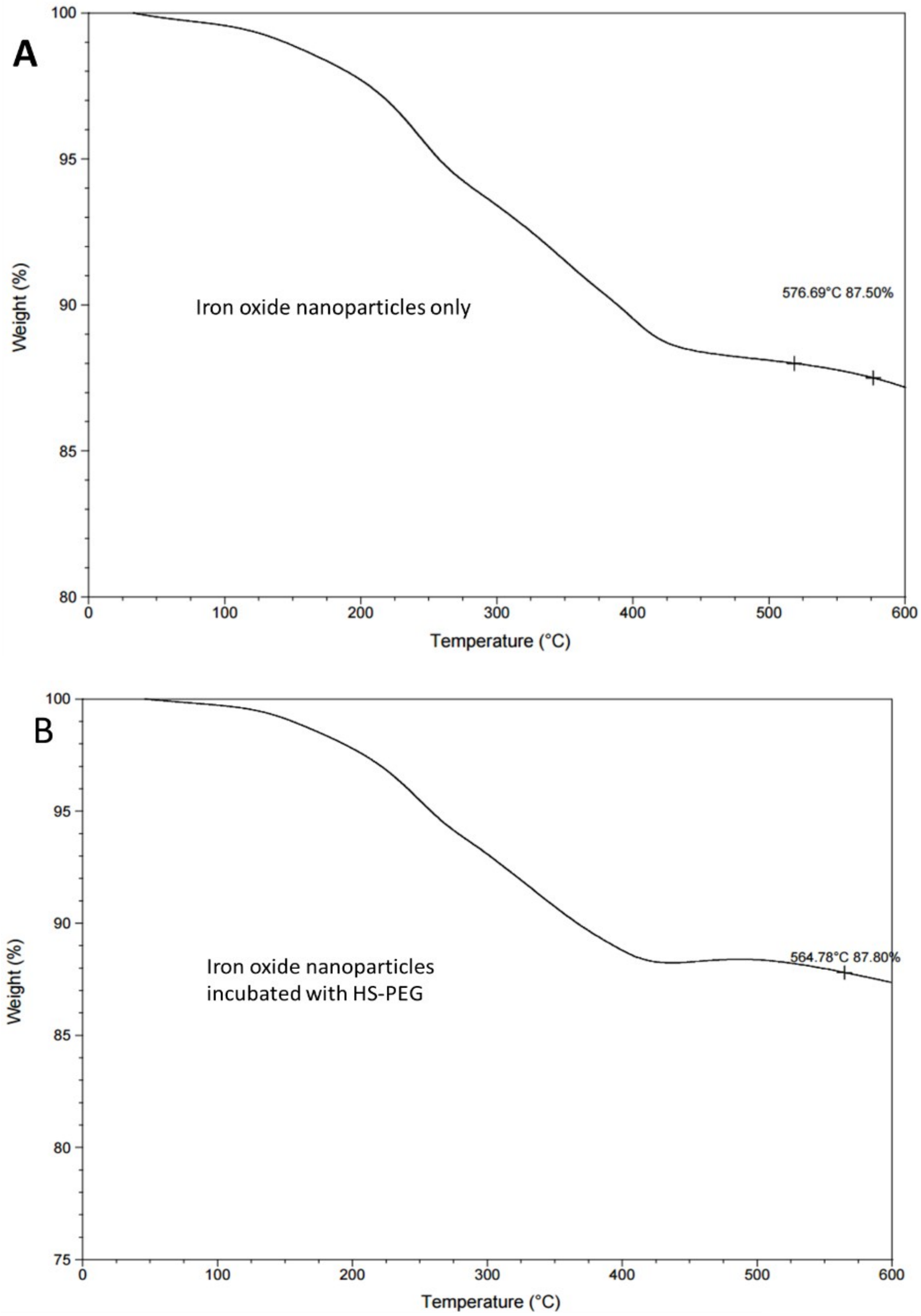

Figure S9. A is the TGA analysis of iron oxide NPs only. B is the TGA analysis of iron oxide NPs incubated with HS-PEG under same conditions as iron oxide-S-PEG.

References

1. Xie, X.; Gao, N.; Deng, R.; Sun, Q.; Xu, Q.; Liu, X. *J. Am. Chem. Soc*. **2013**, 135, 12608-12611.
2. Park, J.; An, K.; Hwang, Y.; Park, J.; Noh, H.; Kim, J.; Park, J.; Hwang, N.; Hyeon, T. *Nat. Mater*. **2004**, 3, 891-895.
3. Schladt, T. D.; Graf, T.; Tremel, W. *Chem. Mater*. **2009**, 21, 3183-3190.
4. Smith, B. C. (1999). *Infrared spectral interpretation: A systematic approach.* Boca Raton: CRC Press.
